# Antimicrobial-Induced DNA Damage and Genomic Instability in Microbial Pathogens

**DOI:** 10.1371/journal.ppat.1004678

**Published:** 2015-03-26

**Authors:** Rebecca S. Shapiro

**Affiliations:** 1 The Broad Institute of MIT and Harvard, Cambridge, Massachusetts, United States of America; 2 Institute of Medical Engineering and Science, Department of Biological Engineering, and Synthetic Biology Center, Massachusetts Institute of Technology, Cambridge, Massachusetts, United States of America; 3 Department of Biomedical Engineering and Center of Synthetic Biology, Boston University, Boston, Massachusetts, United States of America; Geisel School of Medicine at Dartmouth, UNITED STATES

## Introduction

Combatting infectious disease is a critical global health concern and involves tackling both emerging infectious agents and newly–drug resistant strains of previously curable pathogens. The widespread and inappropriate use of antimicrobial agents has increased the frequency of resistance among human pathogens, including bacteria, fungi, and protozoan parasites, and threatened to undermine the efficacy of all existing antimicrobial drugs [1]. Whereas lethal doses of antimicrobials may select for preexisting resistant microbes, there is increasing interest in uncovering the cellular consequences of sublethal antimicrobial exposure on the development of antimicrobial resistance. There are numerous circumstances under which microbial organisms are exposed to low doses of antimicrobials, including in patients, in livestock animals, and in the environment [1–3]. Sublethal antimicrobial exposure can trigger DNA damage and genomic instability across the diversity of microbial pathogens, including bacterial and fungal species.

Here we investigate general mechanisms by which antimicrobials can damage microbial DNA. We also explore downstream cellular responses to DNA damage, including DNA repair. We will look at specific examples by which antimicrobial treatment, through DNA damage and cellular responses, can induce genetic perturbations ranging from small nucleotide mutationsto gross chromosomal rearrangements [1,4]. Overall, this review aims to explore genomic pressure exerted on bacterial and fungal pathogens by antimicrobial treatment, and implications for antimicrobial resistance.

## Antimicrobial-Induced DNA Damage and Repair in Microbial Organisms

**Fig 1:**
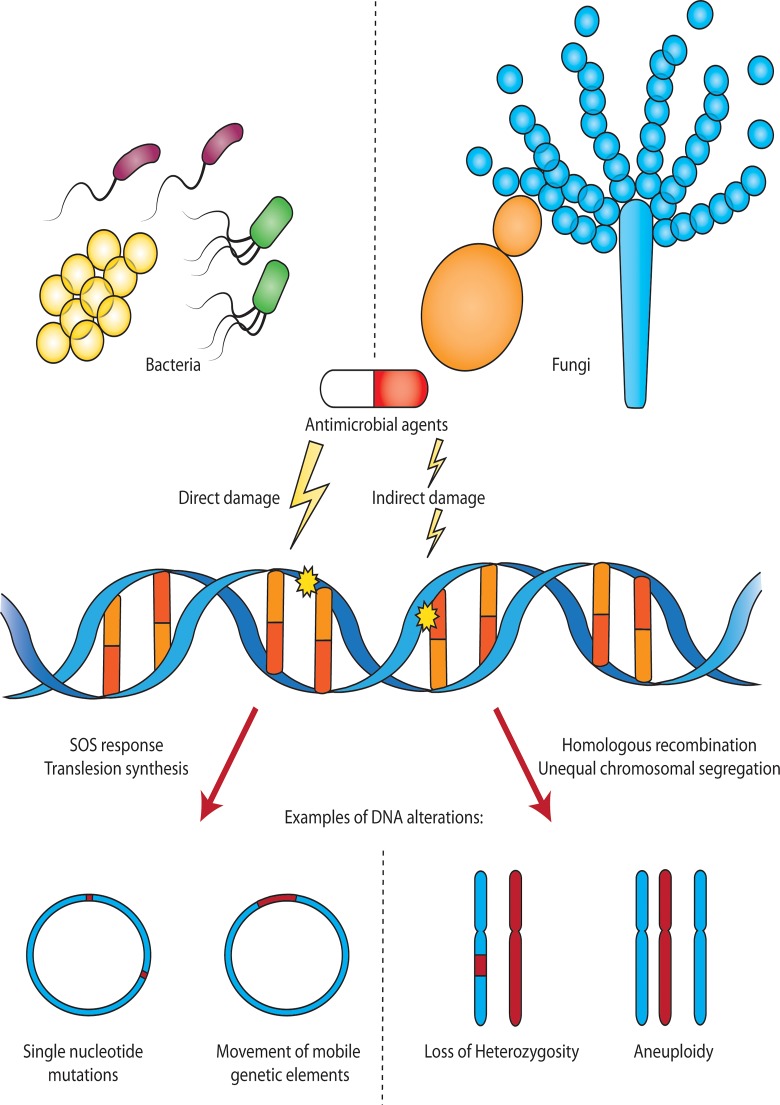
Antimicrobial-induced DNA damage in bacterial and fungal pathogens. Sublethal doses of antimicrobial agents can directly or indirectly damage DNA in bacteria and fungi. In bacteria, DNA damage may lead to up-regulation of an SOS response, error-prone translesion DNA synthesis, or other stress responses that result in mutations including single nucleotide polymorphisms (SNPs) and the movement of mobile genetic elements. In fungi, treatment with antifungals can lead to DNA damage, resulting in homologous recombination and loss of heterozygosity (LOH), or other cellular stress responses, leading to unequal chromosomal segregation during mitosis and aneuploidy. Bacteria and fungi are not to scale.

Microbial species contend with numerous environmental perturbations that can lead to DNA damage, including exposure to direct damage by ultraviolet (UV) light, or damage by chemical compounds. The ability to repair DNA damage and maintain genomic integrity is fundamental to survival of both bacterial and fungal pathogens. Even low doses of antimicrobials can directly or indirectly induce DNA damage and alterations (Fig. 1). In this section, we discuss general mechanisms by which antimicrobials can damage DNA, and strategies employed by microbial species to repair this damage.

DNA damage by antimicrobial agents may occur by several distinct mechanisms. First, several antimicrobial agents cause direct chemical damage to DNA. An example of this is the antibiotic bleomycin, which binds DNA and directly induces double-strand breaks by a mechanism that is not fully understood [5]. Second, antimicrobials may interact with their target protein in a manner that directly induces DNA damage. For instance, the quinolone class of antibiotics specifically inhibits the ligase domain of topoisomerase enzymes, leaving the nuclease domains intact and thereby permitting the enzyme to cut DNA without re-ligation [6]. Finally, numerous antimicrobials result in metabolic perturbations, downstream of the interaction with their respective cellular targets. A commonly observed example of this is the production of reactive oxygen species (ROS) in response to antibiotics (including β-lactams, aminoglycosides, and quinolones) [7–9], antifungals (including polyenes and azoles) [10,11], and antiparasitics [12]. Antimicrobial-induced ROS, such as hydroxyl radicals, damage DNA through the formation of DNA strand breaks, and the incorporation of oxidized guanine residues into the genome [13,14].

Repair of damaged DNA is critical for microbial survival, yet certain DNA damage repair pathways may introduce mutations into the genome. For bacteria, the SOS response is the global response to DNA damage. Triggered by intracellular uncoated single-stranded DNA (ssDNA), the SOS response can be induced upon DNA damage, via the activation of RecA [15]. RecA polymers bind ssDNA, and upon activation, stimulate cleavage of the LexA repressor, leading to derepression of SOS genes, including enzymes involved in DNA repair processes such as nucleotide excision repair or recombination [16]. As part of DNA repair, there can be a trade-off between survival and the fidelity of repair. Thus, bacteria may employ a DNA damage tolerance strategy, where low-fidelity DNA polymerases Pol IV and Pol V are induced and facilitate DNA replication across DNA damage lesions in a manner that introduces errors into the genome [17]. Eukaryotic microbes have homologous strategies to repair or tolerate DNA damage, with a global response involving the expression of genes involved in nucleotide excision repair, and error-prone translesion synthesis polymerases such as DNA polymerase zeta, and Rev1 [18]. In both bacteria and fungi, repair of DNA double-strand breaks may occur through non-homologous end joining, where cut ends are re-ligated in a manner that may be mutagenic, or through homologous recombination, using a homologous sequence as a template for repair [18,19].

## Antimicrobial-Induced Single Nucleotide Mutagenesis

As a result of antimicrobial-induced DNA damage and repair discussed above, as well as additional stress-response pathways, microbial species may experience genomic instability. One example of this is an increase in the number of single nucleotide polymorphisms (SNPs) in response to antimicrobial treatment (Fig. 1). The mechanisms by which this occurs can broadly be categorized into DNA damage response pathways, and other stress response signaling pathways.

In bacterial species, one important pathway that mediates antimicrobial-induced mutagenesis is the DNA damage-induced SOS response. Sublethal doses of diverse classes of antibiotics, including aminoglycosides and quinolones, lead to a cellular SOS response in many bacterial species [20,21]. As described above, quinolones induce DNA damage through interaction with DNA topoisomerase, and thus elicit a bacterial SOS response. In *Escherichia coli*, quinolone-induced SOS leads to derepression of polymerases Pol II, Pol IV, and Pol V, which together introduce mutations into the genome [20,22]. Other classes of antibiotics, such as aminoglycosides, stimulate an SOS response as a result of drug-induced oxidative DNA damage [23]. In *Vibrio cholerae* and *Klebsiella pneumoniae*, SOS-mediated depletion of base excision repair factors such as the mismatch repair protein MutY likely leads to antibiotic-induced mutagenesis upon treatment with aminoglycosides [21,23].

Other general stress response pathways have also been implicated in antimicrobial-induced mutations. In bacteria, the RpoS sigma factor is a central regulator of the general stress response, which is activated in response to stress conditions. In *E*. *coli*, *Pseudomonas aerigunosa*, and *V*. *cholerae*, different classes of antimicrobial agents induce RpoS [23,24]. This leads to activation of the error-prone Pol IV polymerase and down-regulation of accurate DNA repair activity via the mismatch repair protein MutS, thus promoting mutations [24]. Up-regulation of general stress response pathways similarly mediate stress-induced mutations in fungal species. In the model yeast *Saccharomyces cerevisiae*, stress triggers an environmental stress response pathway, mediated through transcriptional regulators Msn2 and Msn4. Similar to what is observed in bacteria, these transcription factors activate downstream error-prone translesion synthesis via the Rev1 polymerase, thus increasing mutagenesis [25]. While antifungal-induced SNP mutagenesis has not been well documented in fungal pathogens, analysis of *S*. *cerevisiae*, with conserved regulatory machinery with pathogenic fungi [26], may provide novel mechanistic insight for fungal pathogens. For both bacterial and fungal pathogens, antimicrobial-induced mutagenesis has the capability to accelerate the acquisition of drug resistance and multi-drug resistance by increasing genetic and phenotypic diversity within the population [27], with important consequences for clinical use of antibiotics.

## Large-Scale Genomic Alterations Induced by Antimicrobial Treatment

In addition to nucleotide mutagenesis, treatment with sublethal antimicrobial agents can also promote larger-scale genomic rearrangements in microbial pathogens. This includes movement of mobile genetic elements, chromosomal rearrangements, and whole chromosome aneuploidies. Such large-scale alterations highlight the difference between bacterial and fungal pathogens. Bacteria are able to exchange genetic information between individual cells via horizontal gene transfer, which occurs far more rarely amongst fungal pathogens [28]. Further, while the genetic material of bacterial pathogens is contained within a limited number of circular chromosomes and plasmids, fungal pathogens typically have several linear chromosomes, and may exist in haploid or diploid states [29]. Such differences in chromosomal number, ploidy, and replication are reflected in the forms of genomic alterations that occur in these pathogens upon antimicrobial treatment.

In bacteria, antimicrobial treatment can trigger the movement of mobile genetic elements [1]. For *Staphylococcus aureus* bacteria, treatment with subinhibitory concentrations of quinolone antibiotics leads to up-regulation of the LexA-dependent SOS response, resulting in increased transposition of the IS256 transposable insertion element [30]. Similarly, antibiotic-induced SOS mediates the movement of integrating conjugative elements (ICEs), a group of bacterial mobile genetic elements that integrate into the chromosome and transfer between cells during conjugation [31]. In *V*. *cholerae* bacteria, sublethal doses of quinolone antibiotic induces an SOS response, which increases the expression of genes necessary for ICE transfer, and thus the frequency of conjugative transfers of this mobile element [31]. As many ICEs encode antibiotic resistance determinants, antibiotic-induced transposition between cells may promote the spread of antibiotic resistance genes [31]. Antibiotics can also stimulate the movement of mobile elements indirectly, by increasing cellular competence [32,33]. For *Streptococcus pneumoniae*, antibiotic-induced genomic replication stress results in stalled replication forks, while DNA replication initiation proceeds [32]. This results in an amplification and overexpression of genes in proximity to the origin of replication, including factors involved in natural cellular competence [32]. This increase in competence and genetic transformability facilitates the acquisition of antibiotic resistance by allowing these pathogens to more readily uptake DNA, including antibiotic resistance determinants, from their environment [32]. Thus antibiotic treatment can both induce movement of antibiotic-encoding mobile elements, and stimulate cellular competence, which together can strongly promote the acquisition and spread of genetic resistance determinants within populations.

Although horizontal gene transfer is rarely observed amongst human fungal pathogens, treatment with antifungals can promote alternative forms of genomic instability, via gross chromosomal rearrangements. The antifungal fluconazole, which targets fungal membrane integrity, also leads to the up-regulation of cellular stress response pathways [29], and promotes genomic rearrangements [4]. For the diploid fungal pathogen *Candida albicans*, sublethal doses of fluconazole promote increased rates of loss of heterozygosity (LOH) [34], a form of gross chromosomal rearrangement in diploid organisms that results in the loss of genetic heterozygosity at a particular locus or throughout an entire chromosome (Fig. 1). Furthermore, *C*. *albicans* exposed to antifungal stress promotes the formation of isochromosomes, in which entire chromosome arms are exchanged, creating a chromosome comprised of two identical chromosome arms flanking a centromere [35]. Although the mechanism of antifungal-mediated chromosomal alterations is unknown, it has been suggested that DNA double-strand breaks induced by antifungal agents [34,36] and repaired via recombination between chromosomes, may contribute to such genomic rearrangements. Both LOH and isochromosomes play an important role in acquired resistance to antifungals in *C*. *albicans*, through homozygosis and duplication of genes encoding both the drug target of the azoles (ergosterol biosynthesis enzyme Erg11), and regulators of drug efflux [4]. Duplication and thus overexpression of Erg11 reduces the efficacy of the azole drugs and promotes resistance, while duplication of transcriptional regulators of drug efflux pumps (such as Tac1 and Mrr1), may promote multi-drug resistance by increasing the efficacy by which antifungals are exported from the cell [4].

Finally, several eukaryotic pathogens have especially plastic genomes, and readily become aneuploid via entire chromosome gains or losses under antimicrobial stress conditions. As aneuploidy results from errors in mitotic cell division, differences between eukaryotic mitosis and binary fission in prokaryotic bacteria, likely accounts for this phenomenon in eukaryotic pathogens. Fungal pathogens, including *C*. *albicans* and *Cryptococcus neoformans*, have particularly flexible genomes [4,37], and aneuploid lineages of these pathogens are frequently identified both in the laboratory, and amongst clinical isolates [38]. The antifungal agent fluconazole induces the formation of aneuploidies in *C*. *albicans* [35,39], and chromosome disomies in *C*. *neoformans* [40,41] (Fig. 1), both of which are linked with the development of antifungal drug resistance from increased copy numbers of key antifungal resistance determinants, including antifungal target proteins and drug transporters [40]. In *Candida* species, this stress-induced aneuploidy occurs from aberrant mitosis due to antifungal stress, resulting in the formation of tetraploid cells, and unequal chromosomal segregation [42]. In *S*. *cerevisiae*, stress-induced aneuploidies occur under diverse stress conditions, including low-dose antifungal treatment, and are linked to protein chaperone Hsp90-mediated disruption of the kinetochore complex, leading to chromosomal instability [43]. Antimicrobial-induced genomic instability leading the chromosomal aneuploidies, including those associated with drug resistance, is a unique way in which fungal pathogens adapt to antimicrobial stress conditions.

## Conclusions

Antimicrobial-induced DNA damage and genomic instability occurs across the diversity of bacterial and fungal pathogens. However, the types of genetic alterations vary between these prokaryotic and eukaryotic pathogens, which differ in their genomic composition, as well as mechanisms of genetic replication and cell division. For instance, although diploid fungal pathogens such as *C*. *albicans* may be buffered against the effects of certain mutations, they are more likely to undergo aneuploidy or LOH events between homologous chromosome pairs. Despite these differences, genetic alterations that are advantageous, including direct genetic alterations that confer antimicrobial resistance, as well as indirect alterations such as increased cellular competence, may facilitate pathogen survival in the face of antimicrobial stress.

The scale of stress-induced genomic alterations, from SNPs to whole chromosome aneuploidy, likely has varying degrees of phenotypic consequences for microbial pathogens. For many fungal pathogens, which unlike bacteria, cannot increase their genetic diversity through horizontal gene transfer, and which rarely undergo sexual reproduction for genetic recombination [44], large-scale chromosomal rearrangements and aneuploidies may provide a unique mechanism to rapidly generate genetic diversity and adapt to their environments under conditions of stress. This mechanism may further extend to other eukaryotic pathogens, such as the trypanosomal parasite *Leishmania*. Like fungal pathogens, stress-induced aneuploidies occur in *Leishmania* in the presence of antiparasitic drugs, potentially as a result of known roles for certain anti-trypanosomal drugs in spindle apparatus formation and chromosome segregation [45]. This suggests that genome plasticity may be conserved across diverse eukaryotic pathogens, and may provide a distinctive mechanism for stress adaptation.

As stress-induced mutation provides a mechanism for microbial pathogens to develop resistance, it is critical to understand how antimicrobial therapeutics may enhance or limit pathogen evolvability. One therapeutic strategy to limit acquired drug resistance is to target the pathogen response to antimicrobials [19]. For instance, preventing SOS induction by targeting central SOS regulators such as the protease LexA can prevent mutations and the evolution of antibiotic drug resistance in *E*. *coli* [22,46]. Similarly, quinolone antibiotics do not induce mutations in *Salmonella typhimurium* strains lacking the Pol V homolog [47]. Additionally, new research has identified certain antimicrobial peptides that, unlike antibiotics, do not elicit an SOS response or increase bacterial mutation rate [48]. This finding suggests promising avenues for identifying novel antimicrobial agents that do not expedite the evolution of antimicrobial resistance.
